# Quantifying numerical and spatial reliability of hippocampal and amygdala subdivisions in FreeSurfer

**DOI:** 10.1186/s40708-023-00189-5

**Published:** 2023-04-07

**Authors:** Isabella Kahhale, Nicholas J. Buser, Christopher R. Madan, Jamie L. Hanson

**Affiliations:** 1 University of Pittsburgh, Pittsburgh, PA USA; 2 University of Nottingham, Nottingham, UK

**Keywords:** Amygdala, Hippocampus, Automated segmentation, FreeSurfer, FreeSurfer 7.1

## Abstract

On-going, large-scale neuroimaging initiatives can aid in uncovering neurobiological causes and correlates of poor mental health, disease pathology, and many other important conditions. As projects grow in scale with hundreds, even thousands, of individual participants and scans collected, quantification of brain structures by automated algorithms is becoming the only truly tractable approach. Here, we assessed the spatial and numerical reliability for newly deployed automated segmentation of hippocampal subfields and amygdala nuclei in FreeSurfer 7. In a sample of participants with repeated structural imaging scans (*N* = *928*), we found numerical reliability (as assessed by intraclass correlations, ICCs) was reasonable. Approximately 95% of hippocampal subfields had “excellent” numerical reliability (ICCs ≥ 0.90), while only 67% of amygdala subnuclei met this same threshold. In terms of spatial reliability, 58% of hippocampal subfields and 44% of amygdala subnuclei had Dice coefficients ≥ 0.70. Notably, multiple regions had poor numerical and/or spatial reliability. We also examined correlations between spatial reliability and person-level factors (e.g., participant age; T1 image quality). Both sex and image scan quality were related to variations in spatial reliability metrics. Examined collectively, our work suggests caution should be exercised for a few hippocampal subfields and amygdala nuclei with more variable reliability.

**Graphical Abstract:**

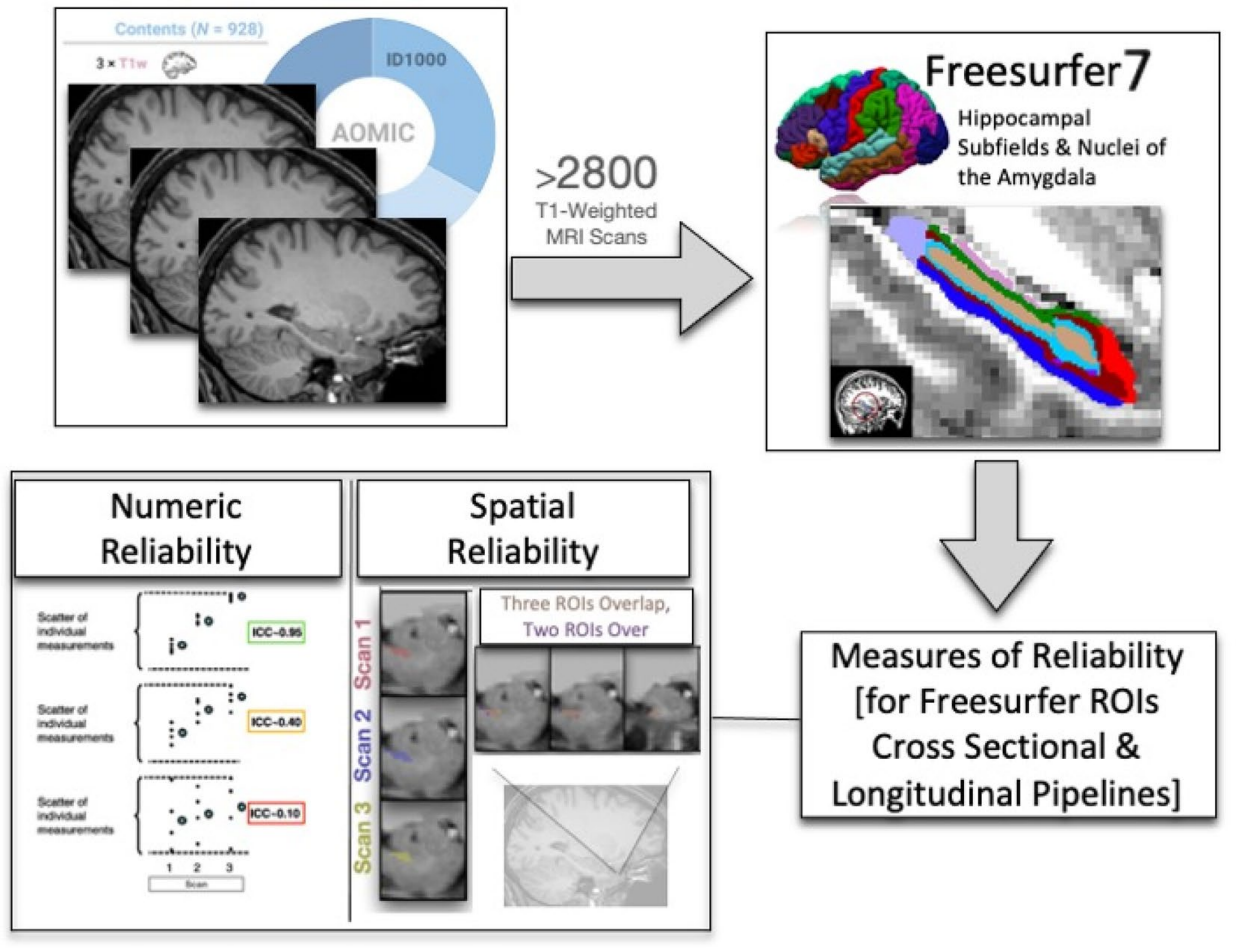

**Supplementary Information:**

The online version contains supplementary material available at 10.1186/s40708-023-00189-5.

## Introduction

The hippocampus and amygdala are brain regions that play key roles in generating and modulating our responses to emotions and stress [2, 66]; they are subsequently two of the most commonly explored and cited brain regions in research. In fact, a query on PubMed revealed that nearly 84,000 publications within the last 10 years alone referenced the hippocampus or the amygdala [1]. A clear understanding of the structure and function of brain regions supporting a variety of emotion-related processes has implications for both psychological well-being and physical health [17]. For example, both the hippocampus and amygdala show volumetric alterations in different neurodegenerative diseases and various forms of psychopathologies, including Alzheimer’s, Major Depression, Anxiety Disorders, and Autism [9, 31, 77, 87]. Continued study of these subcortical structures could further our knowledge on emotions, memory, decision making, and other processes and may lead to novel intervention strategies for different disorders.

Early studies focused on the hippocampus and amygdala typically examined volumes of these regions using expert manual tracing [ 8, 28, 32, 47, 51, 79, 88]. These approaches were at the time necessary to obtain reliable and valid measures of the size of these key brain areas, but hand-tracing is often exceedingly time intensive. As work in this space has continued, large-scale structural MRI-data sets (Ns from 100 to 1000 subjects) are now commonly available [48] and work has shifted from manual tracing of regional volumes. Researchers are now able to leverage ever-improving computational algorithms to automatically segment structural images into their component anatomical structures [49]. These approaches represent a scalable and less demanding method to test relations between volumetric measures of these two structures and psychological variables of interest.

A commonly used software suite, FreeSurfer [20] provides a host of functions for structural MRI processing and analysis, including segmenting subcortical structures. Past work has examined both the validity and reliability of hippocampus and amygdala segmentation in FreeSurfer [41, 49, 57]. One can think of validity as how well an output aligns with “ground-truth” (e.g., comparing FreeSurfer automated amygdala segments to expertly hand-traced volumes), while reliability reflects consistency of outputs (e.g., comparing FreeSurfer automated amygdala from repeated scans of the same person, without consideration of any “ground-truth”). Previous work has found strong reliability for FreeSurfer in terms of hippocampus and amygdala segmentations. Published reports examining test—retest reliability of subcortical volume measures have noted intraclass correlations from FreeSurfer ranging from 0.977 to 0.987 for the hippocampus and 0.806–0.889 for the amygdala [42, 45, 85]. Results considering validity have been more mixed. Work has investigated validity by comparing the spatial and numeric overlap between the volumes produced by FreeSurfer against those produced by expert hand tracing, finding reasonable Dice coefficients for the hippocampus, but lower performance on the amygdala (Hippocampus Dice coefficient = 0.82; Amygdala Dice coefficient = 0.72) [33, 56].

In considering both the hippocampus and amygdala, each of these brain areas are often discussed as unitary structures; however, a large body of basic molecular and cognitive neuroscience research underscores that the hippocampus and amygdala each consist of multiple distinct subregions with different information-processing roles. For example, the hippocampus can be subdivided into the following regions: Dentate Gyrus, critical for pattern separation [60]; Cornu Ammonis (CA) 3, central to pattern completion [29]; CA1, important for input integration from CA3 and entorhinal cortex [6]; and Subiculum, relevant for memory retrieval [72]. Most of the past structural neuroimaging work has combined all these regions, using measures of whole hippocampal volume. This may mean a loss of specificity regarding associations with basic cognitive processes as well as neurobiological alterations seen in different disorders. By examining subcortical structure at a more fine-grain scale, results can be more precisely fit to their root cause and better interpreted considering their theoretical implications.

Responding to this issue, the developers of FreeSurfer have expanded their segmentation methods to include a more granular segmentation of hippocampal subregions [38]. To do this, they combined ultra-high-resolution T1-weighted scans of post-mortem samples with subfields of the hippocampus segmented by hand, to develop an automated algorithm. With this algorithm, there appears to be good numerical reliability and slightly lower spatial reliability for these segments, mirroring the reliability work focusing on the whole hippocampus. Numerical reliability and ICCs are focused on the consistent overall volume size (as indexed by the number of voxels in a region), whereas spatial reliability and the calculation of Dice coefficients assess that the set of voxels classified are the same across both cases. These forms of reliability are typically correlated, but segments could have high numerical reliability but low spatial reliability. In such a case, the same number of voxels are being labelled as a brain region, but the voxels are in fact spatially divergent (and may not be the same brain area). Past work has observed high numerical and moderately high spatial reliability for the hippocampal subfields, reporting ICCs ranging from 0.70 to 0.97 and Dice coefficients ranging from approximately 0.60–0.90 [7, 81]. While we focus on numerical and spatial reliability here, on-going work with manual segmentation procedures continue to develop—that is, different research groups are still working to establish a consensus for how to segment the hippocampus [44, 89].

The amygdala, similarly, has its own subdivisions and the reliability of the automatic segmentation of these subdivisions is still unclear. The FreeSurfer team also expanded their segmentation pipeline to cover a set of subdivisions for the amygdala. The algorithm they employ is trained on manually segmented amygdala nuclei from high-definition 7 Tesla ex-vivo MR images and divides this structure into 9 labelled sub-regions. They applied this segmentation to data sets looking at populations with autism [18] and those at risk for Alzheimer's disease [39] finding significant improvements in pathology detection when this more fined grained view of the amygdala was used in the model [73]. However, direct assessment of numerical and spatial reliability for amygdala subdivisions is limited. Quattrini and colleagues (2020) examined these segments in a modest cohort of individuals (total *N* = *133*) and found reasonable reliability for larger subdivisions (> 200 mm^3^ for the amygdala; > 300 mm^3^ for the hippocampus). This work, however, aggregated across 17 research sites and multiple MRI vendors, deployed a dated version of the software (FreeSurfer 6.0), and typically acquired repeated imaging scans across weeks and months. Given these limitations, the consistency of these segments for a more conventional, single-site study, is still an open question, and it is still unclear whether this fine-grained separation is consistent in the areas that the algorithm is automatically dividing and outputting. Such gaps are critical to fill given that many groups are using these algorithms for applied purposes and reporting differences between clinical and non-clinical populations [55, 90].

Motivated by these facts, we seek to provide an in-depth examination of reliability, both numerically and spatially, for FreeSurfer derived hippocampal and amygdala subdivisions. We leverage an open-access data set of repeated structural scans consisting of a robust sample size (*N* = 928 subjects) that provides precise estimates of reliability variables and unprecedently considers multiple scans to obtain these reliability parameters. Specifically, three repeated structural scans were taken on the same day and same scanner for a total of over 2700 included scans; other similar investigations of reliability have relied on much smaller sample sizes, scans repeated across several days, multiple scanners, and outdated neuroimaging software (e.g., [68]. This investigation minimizes interference from different scanners and benefits from a large sample size, three repeated scans, and up-to-date methods to understand reliability.

In addition to this first-order goal of considering reliability, we also wanted to consider whether person-level (e.g., age, sex) and MR-acquisition (e.g., image quality) factors influence the reliability of these subdivisions. Of note, recent work suggests that MR quality can significantly drive signal variations in structural MRI analyses [27, 49]. Pursuing these aims can inform whether all subdivisions are truly “reliable” and should be explored in FreeSurfer-related analyses, or if caution should be taken in morphometric comparisons (especially for those working in applied areas, e.g., tests of amygdala subdivisions in depressed vs. non-depressed groups). It is critical to highlight less reliable segmentations given the popularity of the hippocampus and amygdala in research and the widespread deployment of FreeSurfer software.

## Methods

### Participants

Data from an open-access neuroimaging initiative, the Amsterdam Open MRI Collection (AOMIC) [76], were used to investigate numerical and spatial reliability of FreeSurfer’s amygdala and hippocampal subregion segmentation algorithms. AOMIC includes structural and functional neuroimaging scans from participants, repeating scans in the same session to see the stability of MRI-based metrics. For this work, data from 928 participants (Average Age = 22.08, Standard Deviation = 1.88) were examined. The majority of participants (*n* = 913, 98% of the sample) had three T1-weighted MR images collected in the same scanning session, while a small subgroup of participants (*n* = 15, ~ 2% of the sample) had two T1-weighted scans. All repeated MRI scans were acquired with the same imaging parameters (noted below).

### MRI scan parameters

MR images were acquired with a Phillips 3 T Intera scanner at the University of Amsterdam. T1-weighted MR images were acquired using a sagittal 3D-MPRAGE sequence (TR/TE = 8.1 ms/3.7 ms, 1mm^3^ voxel, matrix size = 64 × 64). Additional details about the scanning parameters are described by Snoek and colleagues (2021). MRI Images were visually inspected to determine if a participant’s scans should be included in subsequent processing steps (e.g., FreeSurfer).

### Structural neuroimaging processing (FreeSurfer)

Standard-processing approaches from FreeSurfer (e.g., cortical reconstruction; volumetric segmentation) were performed in version 7.1 (Stable Release, May 11, 2020) This was implemented via Brainlife (http://io), a free, publicly funded, cloud-computing platform designed for developing reproducible neuroimaging processing pipelines and sharing data [4, 65]. FreeSurfer is a widely documented and freely available morphometric processing tool suite (http://surfer.nmr.mgh.harvard.edu). The technical details of this software suite are described in prior publications [14, 20–23, 23, 24, 24]. Briefly, this processing includes motion correction and intensity normalization of T1-weighted images, removal of non-brain tissue using a hybrid watershed/surface deformation procedure [75], automated Talairach transformation, segmentation of the subcortical white matter and deep gray matter volumetric structures (including hippocampus, amygdala, caudate, putamen, ventricles), tessellation of the gray matter white matter boundary, and derivation of cortical thickness. Scans from two subjects failed to run to completion in this pipeline and both subjects were removed from further analysis.

FreeSurfer version 7.1 natively includes options to segment hippocampal subfields and amygdala nuclei. The hippocampal segmentation method [37] is based on a hippocampal atlas initially produced from a data set of 15 hand-traced high definition ex-vivo T1-weighted 7 T scans then applied to a set of 39 standard resolution in-vivo MPRAGE scans using parameterized mesh deformations and a probabilistic atlas classification approach. This atlas is used for algorithmic segmentation of MR images pre-processed through the FreeSurfer recon-all pipeline. These images were classified using a parameterized generative model and optimizing the likelihood that any given voxel belongs to the label of a particular hippocampal region in a Bayesian inference framework (for Additional file 1, see [37]. The atlas for this method partitions the hippocampus into the following 12 subfields: (1) Parasubiculum, (2) Presubiculum [Head and Body], (3) Subiculum [Head and Body], (4) CA1 [Head and Body], (5) CA3 [Head and Body], (6) CA4 [Head and Body], (7) Granule Cell and Molecular Layer of the Dentate Gyrus [GC-ML-DG, Head and Body], (8) Molecular layer [Head and Body], (9) Fimbria, (10) Hippocampal Fissure, (11) Hippocampal Tail, and (12) Hippocampus-Amygdala-Transition-Area (HATA. This yields nineteen subdivisions from FreeSurfer (including these regions and head/body divisions.

For the amygdala, the automated segmentation method [73] is based on an atlas produced from 10 hand-traced high definition ex-vivo T1w 7 T scans (5 participants traced bilaterally). As in the hippocampal atlas, this manually segmented ex-vivo data were then applied to the probabilistic classification of the nodes on a parameterized deformation mesh of the amygdala. Similar to the hippocampus, the segmentation of later input data is performed in the framework of Bayesian inference. The amygdala atlas partitions the structure into the following 7 subnuclei: (1) Lateral, (2) Basal, (3) Central, (4) Medial, (5) Cortical, (6) Accessory Basal, (7) Paralaminar. Two additional subdivisions, the Corticoamygdaloid Transition Area and Anterior Amygdaloid Area, are also output.

Of note, here we processed scans from each participant using the “*cross-sectional*” pipeline. This is in contrast to FreeSurfer’s longitudinal stream that creates an unbiased within-subject template image to improve temporal consistency and reduce potential source of bias (e.g., misregistration) [69, 70]. We consider scans processed using FreeSurfer’s “*longitudinal*” pipeline in supplemental analyses (see Additional file 1). Cross-sectional pipelines were applied to the three scans for each participant. For both the hippocampal subfields and amygdala nuclei, volume (in mm^3^ for each subdivision was extracted and used in numerical reliability analysis. Spatial information (labelled voxels in axial, coronal, and spatial orientations was output for each subdivision. Each participant’s T1-weighted scan was then transformed to a common space using FMRIB's Linear Image Registration Tool (degrees of freedom = 6; registering the 2nd and 3rd scans to the participant’s 1st scan). This transformation matrix was then saved and applied to each volume’s labelled output for hippocampal and amygdala subdivisions using a nearest neighbour interpolation; these transformed hippocampal and amygdala subdivisions were then used in spatial reliability analysis.

### Automated MRI image quality assessment

The Computational Anatomy Toolbox 12 (CAT12) toolbox from the Structural Brain Mapping group, implemented in SPM12, was used to generate a quantitative metric indicating the quality of each collected MR image [26]. The method employed considers four summary measures of image quality: (1) noise to contrast ratio, (2) coefficient of joint variation, (3) inhomogeneity to contrast ratio, and (4) root mean squared voxel resolution. To produce a single aggregate metric that serves as an indicator of overall quality, this toolbox normalizes each measure and combines them using a kappa statistic-based framework, for optimizing a generalized linear model through solving least squares [13]. After extracting one quality metric for each scan, we generated three values that represent the difference between two scans (i.e., Scan 1–Scan 2; Scan 1–Scan 3; Scan 2–Scan 3). After taking the absolute value of each of these difference scores, we then averaged them together and used this as a measure of aggregate image quality.

### Derivation of reliability measures

To assess the reliability of numerical volumes output for hippocampus and amygdala subdivisions, we computed intraclass correlation coefficients (ICC) between each labelled sub-region for the test and the retest MRI scans. Of note, an ICC is a descriptive statistic indicating the degree of agreement between two (or more) sets of measurements. The statistic is similar to a bivariate correlation coefficient insofar as it has a range from 0 to 1 and higher values represent a stronger relationship. An ICC, however, differs from the bivariate correlation in that it works on groups of measurements and gives an indication of the numerical cohesion across the given groups [53]. The ICC was calculated separately for each sub-region using the statistical programming language R, with the icc function from the package *‘irr’* [25]. A two-way model with absolute agreement was used to investigate the reliability of subdivision segmentation; this was calculated for each subdivision’s volume (in mm^3^). Although there are no definitive guidelines for precise interpretation of ICCs, results have frequently been binned into three (or four) quality groups, where 0.0–0.5 is “poor”, 0.50–0.75 is “moderate”, 0.75–0.9 is “good” and 0.9–1.0 is “excellent” [10, 43].

In addition to ICCs, Bland–Altman metrics were calculated for each hippocampal and amygdala subdivision using the function blandr.statistics from the package '*blandr'* [15]. In this approach, the mean differences (“*bias*”) between the FreeSurfer outputs (comparing the first and second scan, the first and third scan, and the second and third scan) were first calculated and presented as a portion of the mean volume. We took the absolute value of each of these three values and averaged them together to represent the average Bland–Altman metric across the three scans for a given brain region. Bland–Altman plots were also constructed for a small number of subdivisions to assess agreement between FreeSurfer outputs.

Although ICCs and Bland–Altman metrics serve as indicators of numerical reliability, these may still be incomplete, particularly when we think about the spatial information present in MRI volumes. Indeed, even with numerical similarity, there may be discrepancies in the specific spatial voxels labelled for a given subdivision. To assess whether the voxels assigned to each region were the same between the two timepoints, we calculated the Sørensen-Dice Coefficient using the @DiceMetric program in the AFNI fMRI software package [12]. The Dice coefficient is calculated as (2TP)/(2TP + FP + FN) [TP = True Positive; FP = False Positive; FN = False Negative] and gives an equal weight to criteria of positive predictive value and sensitivity in assessing spatial reliability of subdivisions. Dice coefficients were averaged across the three scans for each brain region to obtain an overall metric of spatial reliability (e.g., one Dice value for the Left Lateral Nucleus, one Dice value for the Right Lateral Nucleus). As recommended by past reports [91, 92], we considered Dice coefficients ≥ 0.700 as exhibiting “good” spatial overlap.

### Statistical analysis

Once overall reliability metrics were calculated, we examined person-level (e.g., age, sex) and MR-acquisition (e.g., MRI quality) factors in relation to these measures. Many different factors may impact amygdala and hippocampal segmentation. For example, past work suggests volumes (of the hippocampus and amygdala) vary with participant age and sex; this association is particularly strong for the hippocampus [16, 61] and suggestive data similarly for the amygdala [51, 52, 64, 67]. Finally, image quality has been shown to have a significant effect on brain volume measurements [27]. Noisier images may lead to gray/white matter misclassification, and impact reliability between different scans. To consider these potential effects, we examined each region's reliability in relation to age, sex, and difference in the CAT12 quality metric. Of note, the average difference in quality between the three scans (described in *Automated MRI Image Quality Assessment*) was included in these analyses.

We computed Pearson’s *r* correlations between Dice coefficients and relevant metrics (i.e., MRI scan quality, sex, and age) using the R function ‘*rcorr’* from package Hmisc (Harrell Jr., 2022). Specifically, for each participant we correlated relevant metrics (the difference in scan quality across 3 scans, sex, and age) with the average of the Dice values across 3 scans for each left and right brain region. We highlighted in Tables 3 and 4 correlations with p values smaller, or equal to, 0.05.

## Results

### Hippocampus reliability

**Fig. 1:**
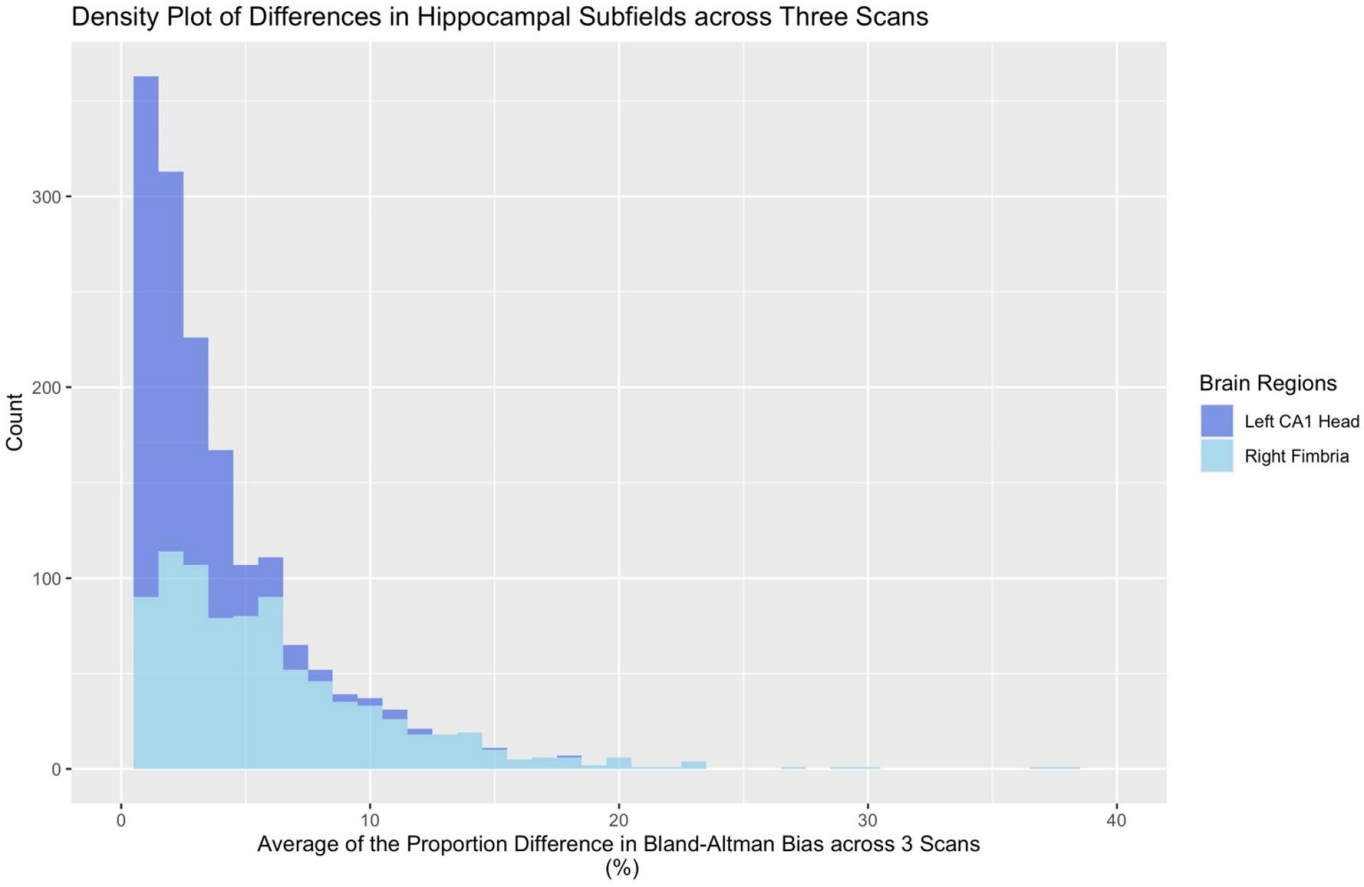
Bland–Altman plots of the average difference for volume estimation across subjects’ three MRI scans for the Left Cornu Ammonis (CA) 1 Head (dark blue) and Right Fimbria (light blue). The horizontal axis indicates the average difference in Bland–Altman “*bias*” (difference between subregional volume output for different scans, as a proportion of a region’s volume), while the vertical axis indicates the number of scans with a given value. Of note, the left CA1 Head has a low degree of mean bias (as a proportion of the region’s volumes; 0.106%), while the right Fimbria has a fair degree of mean bias (1.198%)

Using ICC analysis, we found consistently reasonable levels of numerical reliability for hippocampal subfields. Multiple regions demonstrated “excellent” reliability (ICC ≥ 0.90), while all of the subfields were at least in the “good” range (*ICC* = 0.75–0.90). See Table 1 for values from the 19 subfield segmentations in each hemisphere. Bland—Altman bias indicated some variability with differences between scans, as a portion of that structure’s volume, ranging from 0.078 to 1.198%. See Fig. 1 for a density plot of the average difference in volume estimation across three scans for two hippocampal subfields.

**Table 1:** Intraclass correlation coefficients (ICC), Dice coefficients, Bland–Altman bias as a portion of a volume’s structure (bias as POV), and Bland–Altman bias ranges for Hippocampal Subfields for left and right hemisphere regions (e.g., ICC LH = intraclass correlation coefficients for left hemisphere; Dice RH = Dice coefficient for right hemisphere)

Region	ICC LH	ICC RH	Dice LH	Dice RH	Bias as POV LH (%)	Bias Range LH (%)	Bias as POV RH (%)	Bias Range RH (%)
Parasubiculum	**0.929**	**0.946**	**0.713**	**0.710**	0.356	0.000–45.834	0.410	0.008–32.920
Presubiculum head	**0.924**	**0.936**	**0.792**	**0.792**	0.212	0.001–29.992	0.078	0.002–23.345
Presubiculum body	**0.960**	**0.963**	**0.799**	**0.791**	*1.057*	0.000–41.021	0.626	0.001–33.210
Subiculum head	**0.961**	**0.959**	**0.775**	**0.773**	0.130	0.001–27.278	0.085	0.002–25.029
Subiculum body	**0.961**	**0.964**	**0.823**	**0.826**	0.100	0.004–31.485	0.289	0.002–16.760
CA1 head	**0.971**	**0.979**	**0.818**	**0.825**	0.106	0.000–22.307	0.260	0.000–16.274
CA1 body	**0.948**	**0.970**	**0.751**	**0.780**	0.127	0.001–50.541	0.465	0.001–29.594
CA3 head	**0.952**	**0.969**	*0.662*	*0.675*	0.533	0.000–24.448	0.742	0.000–22.072
CA3 body	**0.933**	**0.950**	*0.597*	*0.627*	0.413	0.000–55.834	0.973	0.001–27.936
CA4 head	**0.966**	**0.966**	**0.793**	**0.800**	0.551	0.000–20.589	0.494	0.000–21.287
CA4 body	**0.931**	**0.938**	**0.767**	**0.780**	0.541	0.002–29.764	0.625	0.002–20.289
GC ML DG head	**0.965**	**0.973**	*0.617*	*0.625*	0.554	0.001–20.329	0.542	0.002–18.378
GC ML DG body	**0.940**	**0.942**	*0.592*	*0.650*	0.500	0.000–21.930	0.503	0.001–27.053
Molecular layer HP head	**0.971**	**0.976**	*0.690*	*0.692*	0.206	0.001–18.600	0.240	0.001–14.279
Molecular layer hp body	**0.954**	**0.961**	*0.631*	*0.647*	0.495	0.001–25.208	0.558	0.000–18.751
Fimbria	**0.936**	**0.942**	*0.681*	*0.679*	0.639	0.008–78.157	*1.198*	0.004–57.572
Hippocampal fissure	*0.887*	*0.888*	0.497	*0.511*	0.443	0.001–41.512	0.586	0.000–45.252
Hippocampal tail	**0.954**	**0.968**	**0.880**	**0.890**	0.315	0.000–50.169	0.393	0.001–18.664
HATA	**0.936**	**0.945**	**0.760**	**0.770**	*1.049*	0.000–34.712	0.216	0.007–26.125
Whole hippocampal body	**0.959**	**0.966**			0.365	0.000–27.935	0.435	0.004–15.245
Whole hippocampal head	**0.975**	**0.980**			0.227	0.001–19.277	0.273	0.002–13.619
Whole hippocampus	**0.976**	**0.983**			0.251	0.001–21.668	0.341	0.000–12.473

Color coding is in accordance with excellent [Bold], good [Italic], poor [Underline] scores for ICCs and Dice coefficients (ICC: 0.90–1.00 [excellent], 0.75–0.89 [good], 0.00–0.74 [poor]; Dice coefficients: 0.70–1.00 [excellent], 0.50–.69 [good], 0–0.49 [poor]). We have also highlighted regions with > 1% bias as a portion of a volume’s structure in Italic

Subfield Abbreviations include: Cornu Ammonis *CA*, Granule Cell and Molecular Layer of Dentate Gyrus *GC-ML-DG*, Hippocampus-Amygdala-Transition-Area *HATA*; Hippocampal Parcellation *HP*

**Fig. 2:**
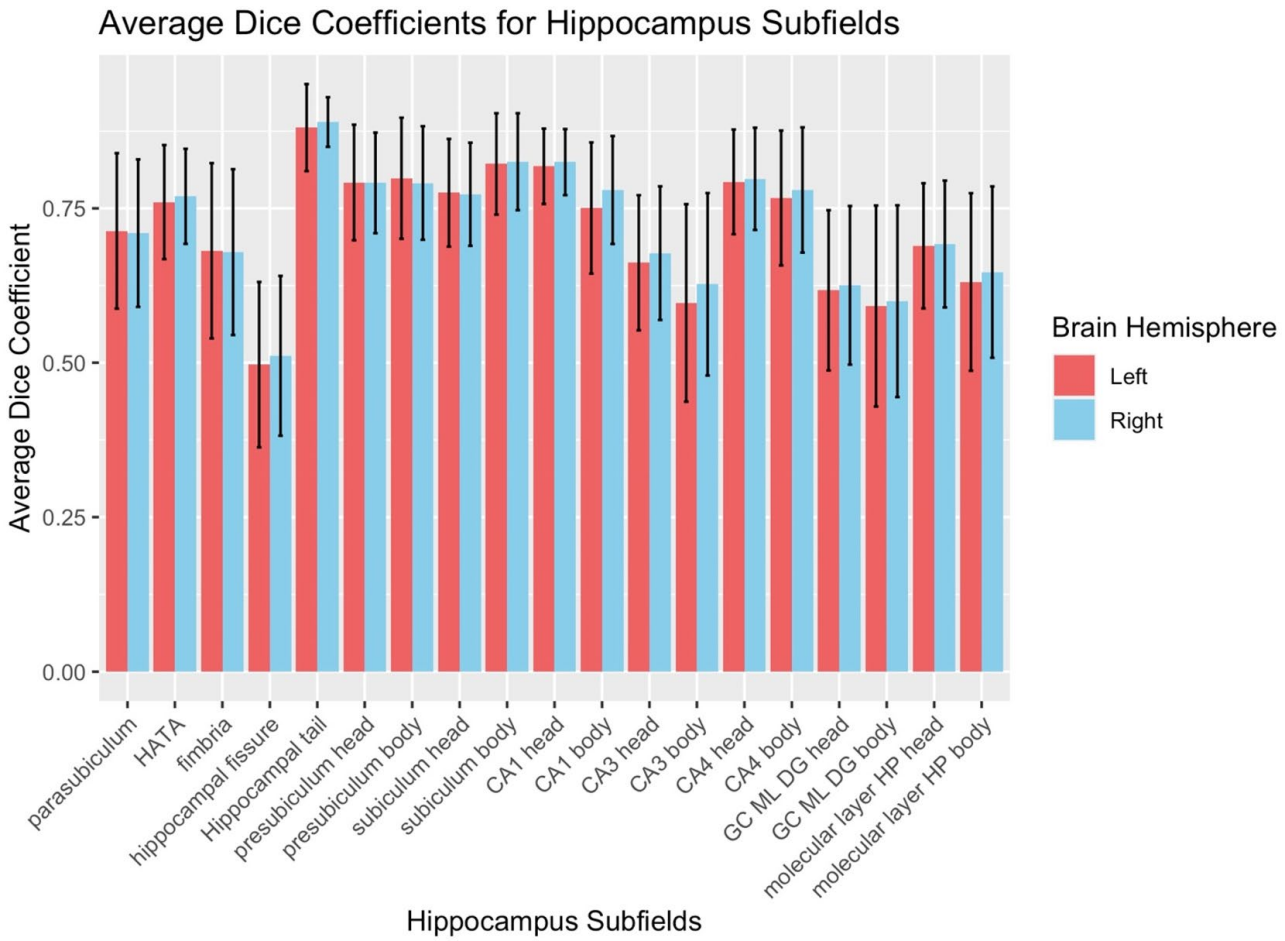
Hippocampal Dice coefficient values for all hippocampal subfields. Error bars represent 1 standard deviation above and below the mean. Subfield Abbreviations include: Cornu Ammonis *CA*, Granule Cell and Molecular Layer of Dentate Gyrus *GC-ML-DG*, Hippocampus-Amygdala-Transition-Area *HATA*, Hippocampal Parcellation *HP*

**Fig. 3:**
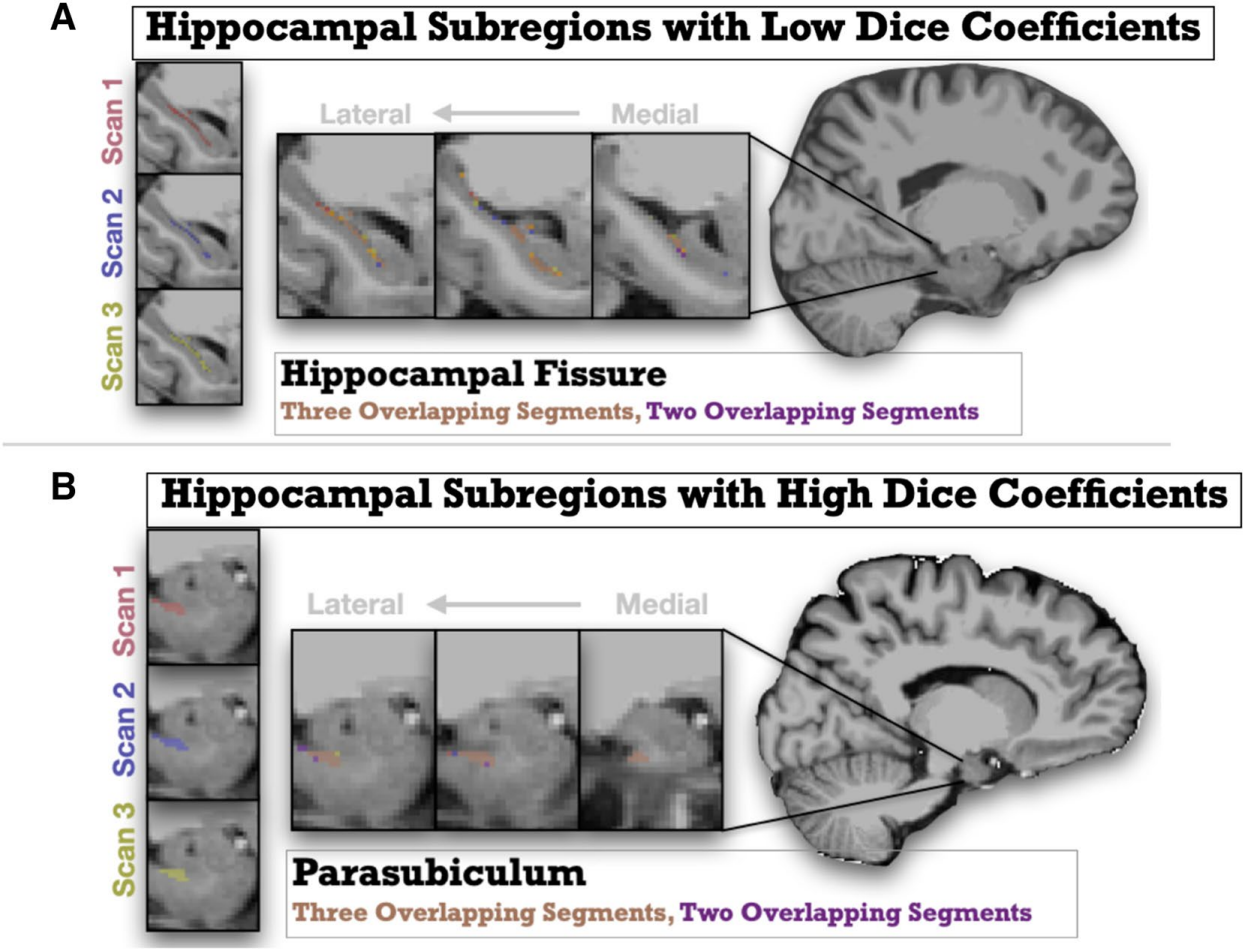
Graphic representations showing magnified depictions of Hippocampal subregions with low and high Dice coefficients (i.e., spatial reliability) from repeated scans (Scan 1 shown in Red; Scan 2 shown in Purple, Scan 3 shown in Yellow). The anatomical (T1w) image underlaid is the unbiased subject template from an example participant. The top panel A represents the hippocampal fissure, an area with low spatial reliability across scans, and the bottom panel B represents the parsubiculum, and area with high spatial reliability. Slices move right to left from medial to lateral

Using Dice coefficients as metrics of spatial reliability, results became a bit more variable with 11 areas showing “excellent” spatial reliability, 7 areas showing “good” spatial reliability, and one area (left Hippocampal fissure) showing poor spatial reliability (Dice coefficient < 0.5). See Fig. 2 for a plot of all Hippocampal Dice coefficient values and Fig. 3 for an example of regions with acceptable spatial reliability (parasubiculum) and poor spatial reliability (hippocampal fissure).

### Amygdala reliability

**Fig. 4:**
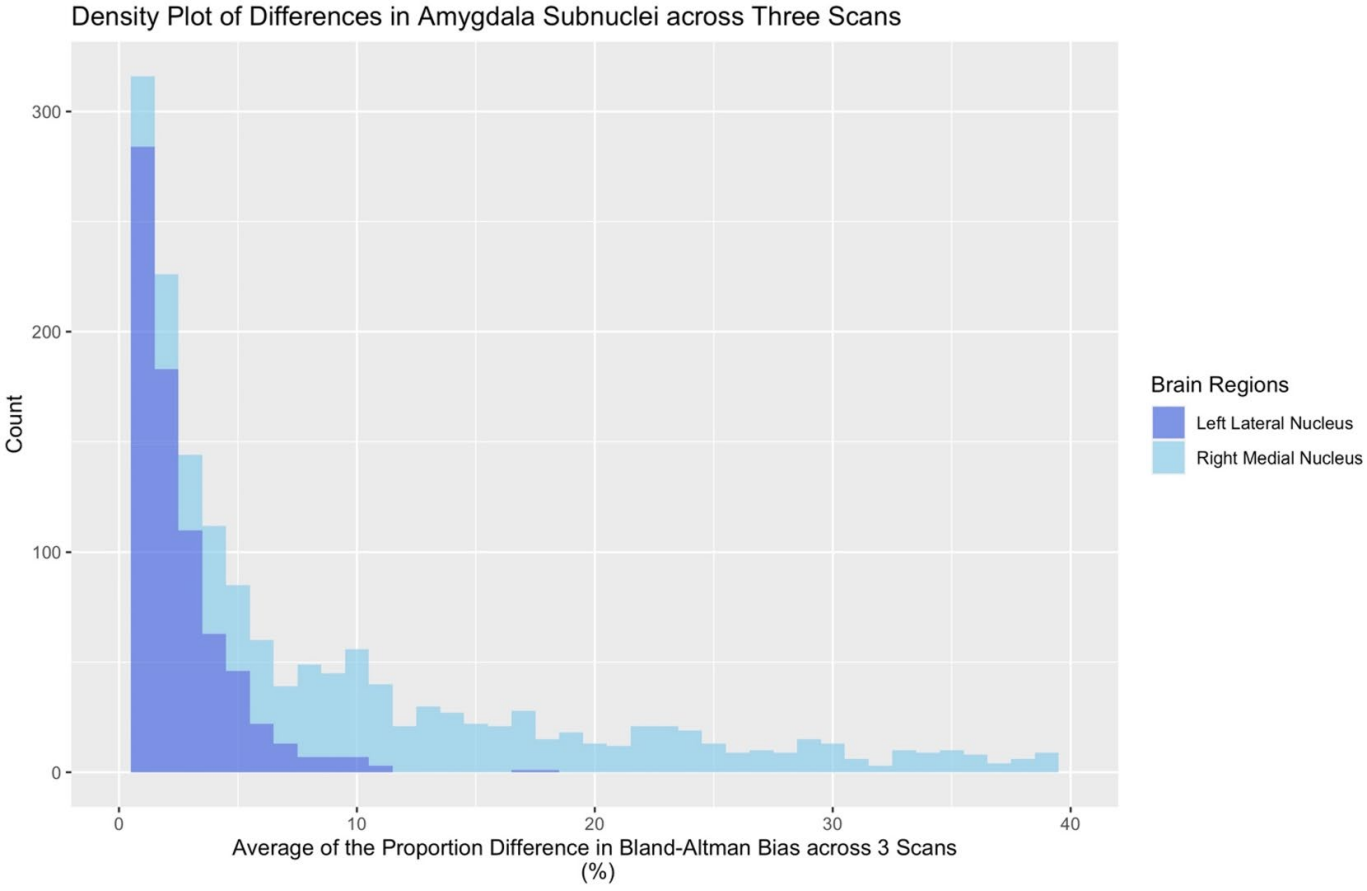
Bland–Altman plots of the average volume difference estimation across Scan 1, Scan 2, and Scan 3 for the left Lateral Nucleus (dark blue) and right Medial Nucleus (light blue). The horizontal axis indicates the average difference in Bland–Altman “*bias*” (difference between subregional volume output for different scans, as a proportion of a region’s volume), while the vertical axis indicates the number of scans with a given value. Of note, the left Lateral Nucleus has a low degree of bias (as a proportion of the region’s volumes; 0.108%), while the right Medial Nucleus has a fair degree of bias (1.047%)

Within the amygdala, the numerical reliability was “excellent” for about 67% of the regions (ICC > 0.90), while the remainder of the regions were in the “good” range (*ICC* = 0.75–0.90) (*see *Table 2). Bland–Altman bias values were somewhat variable with a range of 0.058–1.563%. See Fig. 4 for a density plot of the average difference in volume estimation across three scans for two amygdala subnuclei.

**Table 2:** Intraclass correlation coefficients (ICCs), Dice coefficients, Bland–Altman bias as a portion of a volume’s structure (bias as POV), and Bland—Altman bias ranges for Amygdala Subnuclei for left and right hemisphere regions (e.g., ICC LH = intraclass correlation coefficients for left hemisphere; Dice RH = Dice coefficient for right hemisphere)

Region	ICC LH	ICC RH	Dice LH	Dice RH	Bias as POV LH (%)	Bias range LH (%)	Bias as POV RH (%)	Bias Range RH (%)
Lateral nucleus	**0.964**	**0.956**	**0.900**	**0.899**	0.108	0.003–24.233	0.200	0.001–16.662
Basal nucleus	**0.959**	**0.956**	**0.877**	**0.882**	0.387	0.001–24.086	0.213	0.001–20.245
Central nucleus	*0.895*	*0.867*	*0.600*	*0.607*	0.333	0.007–53.459	0.331	0.002–42.310
Medial nucleus	*0.845*	*0.832*	0.449	0.441	*1.563*	0.004–73.504	*1.047*	0.005–70.814
Cortical nucleus	*0.889*	**0.905**	*0.564*	*0.567*	0.376	0.001–68.640	0.703	0.001–29.931
Accessory basal nucleus	**0.957**	**0.961**	**0.871**	**0.879**	0.224	0.000–21.655	0.647	0.001–16.607
Paralaminar nucleus	**0.941**	**0.946**	0.465	0.480	0.143	0.000–27.786	0.199	0.003–17.924
Corticoamygdaloid transition	**0.939**	**0.949**	**0.758**	**0.760**	0.588	0.002–37.478	0.704	0.005–17.411
Anterior amygdaloid area	**0.901**	*0.858*	*0.630*	*0.641*	0.058	0.006–39.745	0.541	0.003–41.760
Whole amygdala	**0.974**	**0.967**			0.149	0.002–21.679	0.328	0.001–13.837

Color coding is in accordance with excellent [Bold], good [Italic], poor [Undeline] scores for ICCs and Dice coefficients (ICC: 0.90–1.00 [excellent], 0.75–0.89 [good], 0.00–0.74 [poor]; Dice coefficients: 0.70–1.00 [excellent], 0.50–.69 [good], 0–0.49 [poor]). We have also highlighted regions with > 1% bias as a portion of a volume’s structure in Italic

**Fig. 5:**
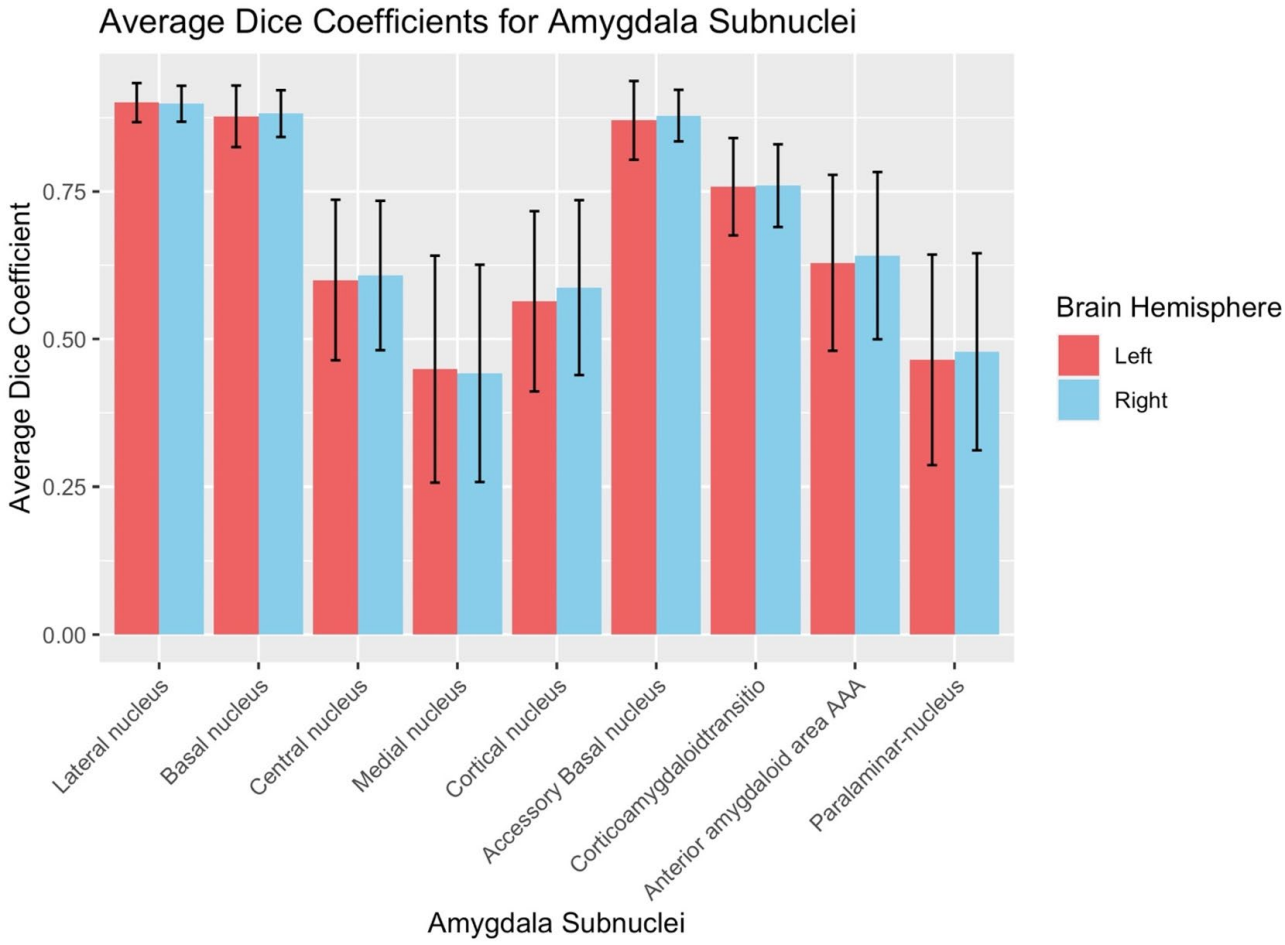
Amygdala Dice coefficient values for all Amygdala subnuclei. Error bars represent 1 standard deviation above and below the mean

**Fig. 6:**
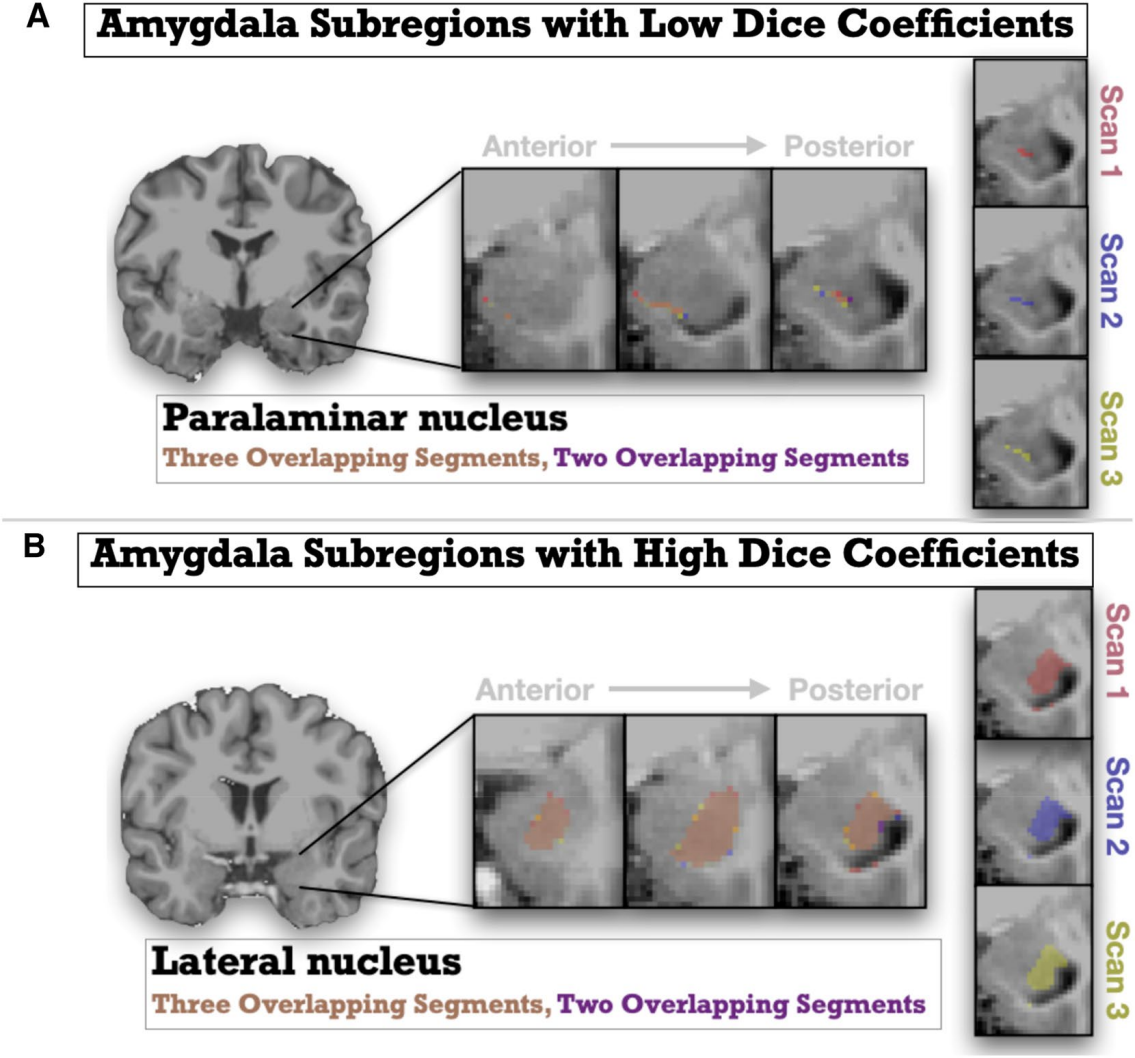
Graphic representations showing magnified depictions of Amygdala subregions with low and high Dice coefficients (i.e., spatial reliability) from repeated scans (Scan 1 shown in Red; Scan 2 shown in Purple, Scan 3 shown in Yellow). The anatomical (T1w) image underlaid is the unbiased subject template from an example participant. The top panel A represents the paralaminar nucleus, an area with low spatial reliability across scans, and the bottom panel B represents the lateral nucleus, and area with high spatial reliability. Slices move right to left from medial to lateral. Multiple slices are depicted left to right, moving anterior to posterior

Regarding spatial reliability, seven areas demonstrated excellent or good reliability (> 0.7) including the lateral, basal, and accessory basal subnuclei (See Table 2). There were, however, areas with poor spatial reliability, including the Medial and Paralaminar Nuclei (Dice Coefficients = 0.30–0.4, See Fig. 5 for a plot of all Amygdala Dice Coefficient values). Figure 6 displays a depiction of the Lateral nucleus, an area with acceptable spatial reliability, and the Paralaminar nucleus, an area with poor spatial reliability.

### Reliability differences in relation to person-level and MR-acquisition factors

We next examined associations between spatial reliability and subject-level variables. Correlations between the Hippocampal-subfield Dice coefficients and our subject-level variables are shown in Table 3. Differences in image quality and participant sex were significantly and negatively related to volumes in a majority of the hippocampal subfields at the *p* < 0.01 level (shown in Table 3). Age was significantly correlated with only a small subset of right hippocampal subfield volumes.

**Table 3:** Correlation coefficients for bivariate correlations between hippocampal subfield Dice coefficients and subject-level covariates: MRI quality (difference score; MRIQ), sex, and age

Region	MRIQ r Dice		Sex r Dice		Age r Dice	
	LH	RH	LH	RH	LH	RH
Parasubiculum	− *0.13*	− *0.08*	− 0.03	− *0.09*	0.00	− 0.05
Presubiculum head	− *0.12*	− *0.16*	− 0.05	− *0.10*	− 0.04	− 0.04
Presubiculum body	− *0.14*	− *0.15*	− *0.08*	− 0.08	− 0.03	0.01
Subiculum head	− *0.11*	− *0.16*	− 0.06	− *0.09*	− 0.03	− 0.05
Subiculum body	− *0.09*	− *0.10*	− 0.06	− *0.09*	0.01	− 0.04
CA1 head	− *0.12*	− *0.14*	− *0.07*	− *0.12*	− 0.06	− 0.05
CA1 body	− 0.03	− *0.07*	− *0.08*	− *0.13*	0.01	− 0.06
CA3 head	− *0.09*	− *0.07*	− *0.09*	− *0.12*	0.00	− 0.06
CA3 body	− 0.05	− *0.08*	− *0.09*	− *0.10*	0.01	− 0.02
CA4 head	− *0.08*	− *0.12*	− *0.08*	− *0.09*	− 0.04	− *0.08*
CA4 body	− 0.04	− *0.11*	− *0.08*	− *0.09*	0.01	− 0.04
GC ML DG head	− *0.10*	− *0.11*	− *0.07*	− *0.10*	− 0.03	− *0.08*
GC ML DG body	− 0.06	− *0.10*	− *0.08*	− *0.10*	0.01	− 0.05
Molecular layer HP head	− *0.13*	− *0.14*	− 0.06	− *0.11*	− 0.05	− *0.07*
Molecular layer HP body	− *0.06*	− *0.10*	− 0.08	− *0.10*	0.02	− 0.05
Fimbria	− *0.11*	− *0.17*	− 0.01	− 0.05	0.02	− 0.02
Hippocampal fissure	− *0.10*	− *0.17*	− *0.08*	− *0.14*	− 0.04	− 0.05
hippocampal tail	− 0.06	− *0.14*	− *0.10*	− *0.11*	− 0.04	− 0.01
HATA	− *0.08*	− *0.10*	− *0.09*	− *0.12*	0.00	− 0.04

These were completed for left hemisphere *LH* and right hemisphere *RH*. Correlations with *p* < 0.05 are highlighted in Italic

Subfield Abbreviations include: Cornu Ammonis *CA*, Granule Cell and Molecular Layer of Dentate Gyrus *GC-ML-DG*, Hippocampus-Amygdala-Transition-Area *HATA,* Hippocampal Parcellation *HP*

Correlations between spatial reliability and subject-level variables for the amygdala nuclei are reported in Table 4. Image quality was significantly and negatively related to a number of regions including the lateral nucleus, the right basal nucleus, the corticoamygdaloid transition, and the right anterior amygdaloid area (at *p* < *0.01*). The spatial reliability of several regions was also significantly and associated with sex and age.

**Table 4:** Correlation coefficients for bivariate correlations between hippocampal subfield Dice coefficients and subject-level covariates: MRI quality (difference score; MRIQ), sex, and age

Region	MRIQ r Dice		Sex r Dice		Age r Dice	
	LH	RH	LH	RH	LH	RH
Lateral nucleus	− *0.11*	− *0.18*	− *0.10*	− *0.11*	0.01	− 0.03
Basal nucleus	− 0.05	− *0.10*	− 0.05	− 0.06	− 0.03	− *0.07*
Central nucleus	− 0.04	0.01	0.04	0.01	− 0.03	− *0.10*
Medial nucleus	− *0.08*	− 0.01	− *0.09*	− *0.06*	0.00	− 0.01
Cortical nucleus	0.00	− 0.04	− 0.06	− 0.04	0.04	− 0.02
Accessory basal nucleus	− 0.04	− *0.07*	− 0.05	− 0.03	− 0.03	− *0.08*
Paralaminar nucleus	− 0.03	− *0.07*	− 0.01	− 0.04	− 0.02	− 0.02
Corticoamygdaloid transition	− *0.10*	− *0.10*	− *0.07*	− *0.07*	− 0.03	− *0.06*
Anterior amygdaloid area	− *0.07*	− *0.13*	− *0.09*	− *0.06*	− 0.03	− 0.05

These were completed for left hemisphere *LH* and right hemisphere (RH). Correlations with *p* < 0.05 are highlighted in Italic

## Discussion

In this paper, we assessed the numerical and spatial reliability of FreeSurfer’s hippocampal and amygdala subdivision segmentation algorithms. The ICCs, serving as our indicator of numerical reliability, were reasonable (hippocampal subfields: 0.887–0.979; amygdala nuclei: 0.832–0.964), indicating that FreeSurfer is generally numerically reliable in providing overall volume for each subregion. Using Bland–Altman metrics of bias as an additional proxy of numerical reliability suggests a few regions exhibited variability in segmentation across scans; specifically, 5 regions across the hippocampus and amygdala showed ≥ 1% bias in volume from one scan to the next. This is concerning given that individuals with dementia (e.g., Alzheimer's disease) or recurrent mental health issues (e.g., depression) often only differ 1–5% from control groups in subcortical volumes (e.g., [40, 46, 74]. The Dice coefficients, serving as our indicator of spatial reliability, were reasonable, though lower than the ICCs. Of potential concern, a few subdivisions in both the hippocampus and amygdala had fairly low spatial reliability, suggesting unreliable segmentation. Examined collectively, applied researchers should take care when applying these types of automated segmentation techniques, especially if not thoroughly trained in amygdala and hippocampal anatomy.

While our results suggest that many of the volumetric outputs of amygdala and hippocampal subdivisions are mostly numerically reliable, the drop in spatial reliability may mean researchers should exercise caution in the analysis and interpretation of areas with poor spatial reliability. For example, the hippocampal fissure, paralaminar nucleus (amygdala), and medial nucleus (amygdala) showed poor spatial reliability (< 0.5) through their Dice coefficients. Because the spatial reliability of these areas is relatively poor, studies that interpret changes in volume within or across subjects might be using segmentations which contain improperly (or inconsistently) classified voxels within those regions. For example, several studies have already reported significant findings from the paralaminar nucleus of the amygdala [55, 90]; given the questionable reproducibility of its anatomical bounding, these findings may require further verification.

Connected to spatial reliability, there are a few potential drivers of the substandard performance in this domain. First, these areas are small and may be difficult to isolate. In such cases, even a few mislabelled voxels can greatly influence spatial overlap. Many of the areas with the lowest spatial reliability are also the smallest subdivisions. For example, the paralaminar and the medial nuclei of the amygdala range between 20 and 60 mm^3^ in our sample and have some of the lowest spatial reliability values. However, this is not the only factor hampering performance, as other structures (of similar sizes) have reasonable spatial reliability values (e.g., HATA ≥ 0.760 Dice coefficients; Parasubiculum ≥ 0.710 Dice coefficients), while comparatively larger structures (e.g., hippocampal-fissure; CA3-body) demonstrate lower spatial reliability. Second, irregular MR contrast is often common to these areas, especially for the amygdala. Given the close vicinity to bone and sinuses, there is typically susceptibility-related dropout, field inhomogeneities, and physiological artifacts in the amygdala and the hippocampus [54, 71, 82]. This may introduce inconsistent gray/white matter contrast, complicating isolation of different subdivisions. Finally, several amygdala and hippocampal subdivisions are irregularly and complexly shaped. For example, both the anterior and posterior borders of the amygdala are difficult to consistently demarcate [1, 11, 19, 80]. Many past reports using manual tracing actually employ “heuristics” rather than clear anatomical boundaries (e.g., [59] used a “semicircle substitution”).

Given these challenges, it is critical to advance novel approaches to segment the hippocampus and amygdala into subdivisions while still maintaining high validity and reproducibility. In regard to the hippocampus, there has been a great deal of progress made by the Hippocampal Subfields Group (HSG); this is a collaboration of > 200 imaging and anatomy experts worldwide that has established guidelines for appropriate MRI acquisition for researchers interested in the hippocampus, as well as developing candidate protocols for the segmentation of hippocampal subregions (eg., [62, 84, 89]. This and other related work have suggested important ways to validate automatic segmentation, including not only comparison to manual delineations, but also replicating known disease effects (e.g., [58]. The HSG and FreeSurfer protocols provide differing guidance on how to subdivide the hippocampus (see Table 3 of [89] for HSG subdivisions). Comparison between HSG and FreeSurfer subdivisions reveals that some hippocampal regions are divided with more granularity according to HSG guidelines, while some regions are more granular with FreeSurfer. For example, while FreeSurfer divides the subiculum, presubiculum, CA1, CA3, CA4, and other regions into “head” and “body,” the HSG does not. Our work suggests that some of these smaller subdivisions may be less reliable than others; Dice coefficients for CA3 head and CA3 body, for instance, were some of the few hippocampal regions in the “good” (and not “excellent”) range. The HSG also includes regions not evaluated by FreeSurfer, including the Entorhinal Cortex, Parahippocampal Cortex, Perirhinal Cortex, while Freesurfer includes regions not considered by the HSG. Importantly, one of the areas identified by our work as having poor reliability—the hippocampal fissure—is not listed as an HSG subdivision. Based on these considerations, we echo HSG’s call to harmonize across protocols, especially across regions with less reliability (e.g., hippocampal fissure), and to re-evaluate some FreeSurfer subdivisions.

Similar joint efforts to HSG are not, to our knowledge, currently underway for amygdala subnuclei segmentation. Convening such a collaborative could be particularly impactful moving forward, especially as debate has been fairly continuous regarding subdivisions of the amygdala at the histological level (e.g., [78]. Our results found that, across both the hippocampus and amygdala, most regions found to have less satisfactory reliability were in the amygdala. Given the popularity of the amygdala as a brain region (e.g., recent reports have found that manuscripts findings on the amygdala are more likely to be published in high-impact journals; [5] and widespread deployment of FreeSurfer by basic and applied researchers, we recommend caution in interpreting findings regarding amygdala subnuclei.

In the interim or the absence of joint efforts to establish more reliable amygdala segmentations, we have several suggestions for higher quality research. It may be reasonable to only consider more macro-level amygdala segmentation (e.g., basolateral, centromedial, basomedial, and amygdaloid cortical complexes, as detailed by [50]. Many groups have moved towards this idea, aggregating subdivisions using latent factor modelling and other techniques to group related regions (e.g., [63]. There is, however, ongoing debate about specific best practices, as even established guidelines for MRI acquisition or landmark in in-vivo data may present additional unforeseen challenges (e.g., Special hippocampal acquisitions providing incomplete coverage of target structure; In-vivo MRI does not supply enough features to define many hippocampal subfield boundaries). In addition, findings suggest that magnetic resonance images with 1 × 1 × 1 1 mm^3^ resolution are too low in quality for investigations of hippocampal subfields [83]; future work should, therefore, strive to use higher resolution images with FreeSurfer segmentation (i.e., resolution smaller than 1 × 1 × 1 mm^3^). Finally, single modality structural imaging (e.g., a T1 scan without a T2 scan) is likely less reliable; protocols could require routines to have multiple imaging modalities or restrict output to more reliable regions if input contains a single imaging modality.

As noted in our introduction, our findings only speak to the reliability of these measures, and not the validity of these segments. Investigations of validity require comparison of automated output with “ground-truth” data typically derived from hand-tracing. Given that our data set contains over 2700 scans (and, therefore, the same number of amygdalae that would require tracing), such an endeavor is less practical for this particular sample. Future work should establish the validity of these FreeSurfer subnuclei divisions, particularly considering the popularity of the amygdala as a brain region [5] and FreeSurfer as a segmentation software. Previous work has compared FreeSurfer’s hippocampal subfields to hand drawn volumes [35, 86]; however, there is yet to be any comparison of automated amygdala subnuclei segmentation to hand-tracing. Reliable methods exist for expert manual segmentation of the amygdala [3, 19, 36]; however, this typically requires high-resolution and high-field strength neuroimaging (i.e., > 3 T MRI Scanner, sub-millimeter voxels). Studies looking at the degree of overlap between such methods and the FreeSurfer algorithm for amygdala segmentation would be helpful for effective evaluation of validity.

Our goal was to present reliability analyses that would be the most relevant to the ‘typical’ structural study, where a T1-weighted single scan is acquired for each participant, recruited from a specific local area. In considering this approach, it is important to note a few potential limitations of our work. First, we processed test—retest MRI images using the cross-sectional FreeSurfer pipeline; we report in our Supplementary Materials using the longitudinal stream. Results were largely consistent across the two pipelines, with several hippocampal subfields and amygdala subnuclei demonstrating *decreased* numeric reliability with the longitudinal processing stream. Furthermore, the following regions were consistently highlighted as having less-than-excellent spatial reliability across both streams: in the hippocampus, the hippocampal fissure, and in the amygdala, the central, cortical, paralaminar, and medial nuclei. We present results from the cross-sectional processing pipeline, as is done in other studies of reliability (e.g., [49], because FreeSurfer’s longitudinal pipeline is not independently segmenting the different MRI scans. This violates some theoretical aspects of test—retest reliability and would be expected to produce a more favorable set of reliability metrics for FreeSurfer’s methods.

Second, we only used T1-weighted scans in FreeSurfer, but additional MRI volumes (e.g., T2-weighted) from the same subjects may yield a more reliable segmentation. FreeSurfer’s developers have worked to allow the amygdala and hippocampal subdivision routines to accept high-resolution T2-weighted volumes, and this should be investigated in future work. Third, the sample is a rather homogenous group of individuals and may not represent the greater population. All participants were recruited from the University of Amsterdam, with limited racial and ethnic variability. Similarly, all participants were neurotypical young adults in a constrained age range (Mean = 22.08 ± 1.88). Additional work considering reliability of the method in a diverse set of populations (e.g., pediatric, elderly, mild cognitive impairment) would be helpful in ascertaining how well these findings generalize outside of our sample population. Finally, this analysis focused on the reliability of FreeSurfer 7.1, since this reflects a more current version of the software. Future work could consider comparing segmentations derived from current FreeSurfer versions with past versions of this software to facilitate our understanding across extant and emerging literature. Recently published work reflects an analogous endeavor for non-segmented brain regions (e.g., the whole amygdala), highlighting key anatomical areas that were less compatible across FreeSurfer software versions 5.3, 6.0, and 7.1 [30]. These authors found good-to-excellent reliability across software versions for subcortical regions (including the hippocampus and amygdala) and reported that FreeSurfer version 7.1 was generally advantageous over earlier versions.

Limitations notwithstanding, our work extends the information provided by previous publications regarding the reliability of FreeSurfer’s subcortical segmentation for the hippocampus, amygdala, and their respective subregions. To our knowledge, this is the first work to directly investigate the test—retest reliability of the amygdala nuclei algorithm in FreeSurfer 7. The strengths of our work include a large sample size, the use of FreeSurfer’s more robust longitudinal pipeline, and the report of mathematically rigorous measures of reliability. Our work provides additional confidence in interpreting those regions with high reliability and a necessary caution in interpretation of those with poorer results.

## Supplementary Information

**Additional file 1: Table S1**: Intraclass Correlation Coefficients (ICC), Dice Coefficients, Bland–Altman bias as a portion of a volume’s structure (Bias as POV), and Bland-Altman biasranges for Hippocampal Subfields for left and right hemisphere regions (e.g., ICC LH =Intraclass Correlation Coefficients for left hemisphere; Dice RH = Dice Coefficient forright hemisphere). Color coding is in accordance with excellent [green], good [yellow], poor [red] scores for ICCs and Dice Coefficients (ICC: 0.90-1.00 [excellent], 0.75-0.89 [good], 0.00-0.74 [poor]; Dice Coefficients: 0.70-1.00 [excellent], 0.50-.69 [good], 0-0.49 [poor]). We have also highlighted regions with >1% bias as a portion of a volume’s structure in yellow. Subfield Abbreviations include: Cornu Ammonis (CA), Granule Cell and Molecular Layer of Dentate Gyrus (GC-ML-DG); Hippocampus-Amygdala-Transition-Area (HATA); Hippocampal Parcellation (HP). Values were derived using Freesurfer 7 longitudinal processing stream. **Table S2**: Intraclass Correlation Coefficients (ICCs), Dice Coefficients, Bland–Altman bias as a portion of a volume’s structure (Bias as POV), and Bland-Altman bias ranges for Amygdala Subnuclei for left and right hemisphere regions (e.g.,ICC LH = Intraclass Correlation Coefficients for left hemisphere; Dice RH = Dice Coefficient for right hemisphere). Color coding is in accordance with excellent [green], good [yellow], poor [red] scores for ICCs and Dice Coefficients (ICC: 0.90-1.00 [excellent], 0.75-0.89 [good], 0.00-0.74 [poor]; Dice Coefficients: 0.70-1.00 [excellent], 0.50-.69 [good], 0-0.49 [poor]). We have also highlighted regions with >1% bias as a portion of a volume’s structure in yellow. Values were derived using Freesurfer 7 longitudinal processing stream. **Table S3**: Correlation coefficient for bivariate correlations between Hippocampal Subfield Dice Coefficients and subject-level covariates: MRI Quality (Difference Score; MRIQ), Sex, and Age. These were completed for left hemisphere (LH) and right hemisphere (RH). Correlations with p<0.05 are highlighted in yellow. Subfield Abbreviations include: Cornu Ammonis (CA), Granule Cell and Molecular Layer of Dentate Gyrus (GC-ML-DG); Hippocampus-Amygdala-Transition-Area (HATA); Hippocampal Parcellation (HP). Values were derived using Freesurfer 7 longitudinal processing stream. **Table S4**: Correlation coefficient for bivariate correlations between Hippocampal Subfield Dice Coefficients and subject-level covariates: MRI Quality (Difference Score; MRIQ), Sex, and Age. These were completed for left hemisphere (LH) and right hemisphere (RH). Correlations with p<0.05 are highlighted in yellow. Values were derived using Freesurfer 7 longitudinal processing stream. **Table S5**: Mean and standard deviation (SD) volume estimates across three scans for each Hippocampal Subfield (LH = Left Hemisphere, RH = Right Hemisphere) in millimeters cubed (mm3). Subfield abbreviations include: Cornu Ammonis (CA), Granule Cell and Molecular Layer of Dentate Gyrus (GC-ML-DG); Hippocampus-Amygdala-Transition-Area (HATA); Hippocampal Parcellation (HP). Volume estimates derived using the FreeSurfer 7 longitudinal processing pipeline **Table S6**: Mean and standard deviation (SD) volume estimates across three scans for Amygdala Subnuclei (LH = Left Hemisphere, RH = Right Hemisphere) in millimeters cubed (mm3). Volume estimates derived using the FreeSurfer 7 longitudinal processing pipeline.

## Data Availability

Neuroimaging data used in our analyses were sourced from the Amsterdam Open MRI Collection (AOMIC, [76]. FreeSurfer software is publicly and freely available from the FreeSurferWiki resource (http://surfer.nmr.mgh.harvard.edu/fswiki/FreeSurferWiki), which is developed and maintained at the Martinos Center for Biomedical Imaging (http://www.nmr.mgh.harvard.edu/martinos/noFlashHome.php). This software, information and support are provided online at the FreeSurferWiki webpage.
