# Supplementary material for: Quantifying numerical and spatial reliability of hippocampal and amygdala subdivisions in FreeSurfer
Source: Brain Inform. 2023 Apr 7;10(1):9. doi: 10.1186/s40708-023-00189-5 (PMC10082143; doi:10.1186/s40708-023-00189-5)
Supplement: Supplementary file 1 — Additional file 1: Table S1: Intraclass Correlation Coefficients (ICC), Dice Coefficients, Bland–Altman bias as a portion of a volume’s structure (Bias as POV), and Bland-Altman biasranges for Hippocampal Subfields for left and right hemisphere regions (e.g., ICC LH =Intraclass Correlation Coefficients for left hemisphere; Dice RH = Dice Coefficient forright hemisphere). Color coding is in accordance with excellent [green], good [yellow], poor [red] scores for ICCs and Dice Coefficients (ICC: 0.90-1.00 [excellent], 0.75-0.89 [good], 0.00-0.74 [poor]; Dice Coefficients: 0.70-1.00 [excellent], 0.50-.69 [good], 0-0.49 [poor]). We have also highlighted regions with >1% bias as a portion of a volume’s structure in yellow. Subfield Abbreviations include: Cornu Ammonis (CA), Granule Cell and Molecular Layer of Dentate Gyrus (GC-ML-DG); Hippocampus-Amygdala-Transition-Area (HATA); Hippocampal Parcellation (HP). Values were derived using Freesurfer 7 longitudinal processing stream. Table S2: Intraclass Correlation Coefficients (ICCs), Dice Coefficients, Bland–Altman bias as a portion of a volume’s structure (Bias as POV), and Bland-Altman bias ranges for Amygdala Subnuclei for left and right hemisphere regions (e.g.,ICC LH = Intraclass Correlation Coefficients for left hemisphere; Dice RH = Dice Coefficient for right hemisphere). Color coding is in accordance with excellent [green], good [yellow], poor [red] scores for ICCs and Dice Coefficients (ICC: 0.90-1.00 [excellent], 0.75-0.89 [good], 0.00-0.74 [poor]; Dice Coefficients: 0.70-1.00 [excellent], 0.50-.69 [good], 0-0.49 [poor]). We have also highlighted regions with >1% bias as a portion of a volume’s structure in yellow. Values were derived using Freesurfer 7 longitudinal processing stream. Table S3: Correlation coefficient for bivariate correlations between Hippocampal Subfield Dice Coefficients and subject-level covariates: MRI Quality (Difference Score; MRIQ), Sex, and Age. These were completed for left hemisphere (L [file 40708_2023_189_MOESM1_ESM.pdf]

Additional file: Quantifying Numerical and Spatial Reliability of Amygdala and Hippocampal Subdivisions in FreeSurfer

Isabella Kahhale, Nicholas J Buser, Christopher R. Madan, & Jamie L. Hanson

The main analyses in our manuscript process the scans using the Freesurfer 7 cross-sectional pipeline; here, we consider Dice coefficients, ICCs, and Bland-Altman statistics from scans processed using the longitudinal stream. The longitudinal processing stream takes results from all time points of the cross-sectional analysis to derive a within-subject template that is used in further processing stages. This method has been shown to reduce variability and avoid over-regularization in processing steps (see Reuter et al., 2012 for more details).

Results

Hippocampus Subfield Reliability using Longitudinal Pipeline

Using ICC analysis, several hippocampal subfields demonstrated decreased reliability when analyzed using the longitudinal processing stream compared to the cross-sectional processing stream. These include the following bilateral subfields: parasubiculum, presubiculum head, CA4 body, GC ML DG body, and HATA. The hippocampal fissure (bilaterally) was highlighted as an area with less-than-excellent reliability across both processing streams.

Dice coefficients processed using the Freesurfer Longitudinal pipeline demonstrated greater spatial reliability compared to Dice coefficients processed using the Cross-sectional pipeline. 18 of the 19 regions considered showed “excellent” spatial reliability, while 1 region (the hippocampal fissure, both left and right) showing poor

spatial reliability (Dice Coefficient<0.5). Notably, this region had consistently poor spatial reliability across both the longitudinal and cross-sectional pipeline. See Table S1 for the DICE coefficients, ICCs, and Bland-Altman statistics for Hippocampal subfields processed using the longitudinal pipeline.

Table S1.

Region	ICC LH	ICC RH	Dice LH	Dice RH	Bias as POV LH	Bias Range LH	Bias as POV RH	Bias Range RH
Parasubiculum	0.860	0.871	0.799	0.795	0.294%	0.000 - 52.632%	0.897%	0.000 - 50.340%
Presubiculum Head	0.892	0.864	0.865	0.855	0.404%	0.000 - 40.486%	0.378%	0.000 - 25.830%
Presubiculum Body	0.940	0.940	0.892	0.876	0.860%	0.000 - 36.620%	0.605%	0.000 - 33.210%
Subiculum Head	0.937	0.923	0.860	0.852	0.457%	0.000 - 48.235%	0.663%	0.000 - 28.179%
Subiculum Body	0.950	0.929	0.902	0.896	0.586%	0.000 - 26.087%	0.089%	0.000 - 27.571%
CA1 Head	0.973	0.974	0.871	0.872	0.424%	0.000 - 13.534%	0.107%	0.000 - 13.873%
CA1 Body	0.946	0.944	0.843	0.851	0.714%	0.000 - 36.015%	1.044%	0.000 - 32.461%
CA3 Head	0.916	0.925	0.762	0.765	0.533%	0.000 - 37.890%	1.114%	0.000 - 33.166%
CA3 Body	0.931	0.929	0.749	0.759	0.907%	0.000 - 47.909%	0.501%	0.000 - 47.059%
CA4 Head	0.908	0.909	0.871	0.872	0.591%	0.000 - 32.813%	0.619%	0.000 - 24.516%
CA4 Body	0.855	0.865	0.885	0.877	0.974%	0.000 - 29.046%	0.682%	0.000 - 33.766%
GC ML DG Head	0.904	0.914	0.737	0.740	0.561%	0.000 - 28.085%	0.440%	0.000 - 25.882%
GC ML DG Body	0.842	0.828	0.750	0.735	0.918%	0.000 - 29.333%	0.454%	0.000 - 31.788%
Molecular Layer HP Head	0.931	0.926	0.782	0.779	0.476%	0.000 - 27.397%	0.264%	0.000 - 17.778%
Molecular Layer HP Body	0.895	0.909	0.771	0.763	0.266%	0.000 - 25.201%	0.595%	0.000 - 23.768%
Fimbria	0.918	0.896	0.783	0.767	1.201%	0.000 - 56.209%	0.963%	0.000 - 76.289%
Hippocampal Fissure	0.796	0.779	0.583	0.587	0.876%	0.000 - 92.754%	2.070%	0.000 - 86.667%
Hippocampal Tail	0.973	0.974	0.928	0.929	0.399%	0.000 - 22.446%	0.491%	0.000 - 14.022%
HATA	0.882	0.889	0.822	0.825	1.046%	0.000 - 46.154%	0.489%	0.000 - 34.286%

Additional file Table S1: Intraclass Correlation Coefficients (ICC), Dice Coefficients, Bland–Altman bias as a portion of a volume's structure (Bias as POV), and Bland-Altman bias ranges for Hippocampal Subfields for left and right hemisphere regions (e.g., ICC LH = Intraclass Correlation Coefficients for left hemisphere; Dice RH = Dice Coefficient for right hemisphere). Color coding is in accordance with excellent [green], good [yellow], poor [red] scores for ICCs and Dice Coefficients (ICC: 0.90-1.00 [excellent], 0.75-0.89 [good], 0.00-0.74 [poor]; Dice Coefficients: 0.70-1.00 [excellent], 0.50-.69 [good], 0-0.49 [poor]). We have also highlighted regions with >1% bias as a portion of a volume's structure in yellow. Subfield Abbreviations include: Cornu Ammonis (CA), Granule Cell and Molecular Layer of Dentate Gyrus (GC-ML-DG); Hippocampus-Amygdala-Transition-Area (HATA); Hippocampal Parcellation (HP). Values were derived using Freesurfer 7 longitudinal processing stream.

Amygdala Subnuclei Reliability using Longitudinal Pipeline

A processing error with the Freesurfer longitudinal pipeline occurred for a few brain regions for a subset of participants. This was likely due to the small size of these particular regions. This error effected the following brain regions and subject counts: Left Anterior Amygdaloid Area (n = 73), Right Anterior Amygdaloid Area (n = 70), Left Medial Nucleus (n = 21). The “bias as percent of variance” and “Bland-Altman bias ranges” for the 3 aforementioned brain regions were calculated with the erroneous subjects missing.

Amygdala subnuclei with poor reliability using ICC metrics were relatively consistent across the two processing streams; however, ICC values were lower for regions computed with the longitudinal processing stream. These included the following bilateral subnuclei: central nucleus, medial nucleus, paralaminar nucleus, and the Anterior Amygdaloid Area. The cortical nucleus (bilaterally) was highlighted as an area with poor reliability *only* when using the longitudinal processing stream.

Mirroring results in our main manuscript using the cross-sectional pipeline, more subregions of the amygdala demonstrated decreased spatial reliability. Five regions demonstrated “excellent” reliability, while 3 demonstrated “good” reliability (central, cortical, and paralaminar nuclei) and 1 demonstrated “poor” reliability (the medial nucleus) (see Table S3 for Dice values for all amygdala subregions). The regions with less-than-excellent reliability demonstrated similar spatial reliability across the cross-sectional and longitudinal processing streams.

Table S2.

Region	ICC LH	ICC RH	Dice LH	Dice RH	Bias as POV LH	Bias Range LH	Bias as POV RH	Bias Range RH
Lateral Nucleus	0.966	0.956	0.929	0.924	0.295%	0.000 - 18.435%	0.270%	0.000 - 18.848%
Basal Nucleus	0.954	0.944	0.926	0.923	0.243%	0.000 - 20.977%	0.367%	0.000 - 14.177%
Central Nucleus	0.807	0.788	0.678	0.675	0.286%	0.000 - 50.000%	0.123%	0.000 - 52.874%
Medial Nucleus	0.596	0.583	0.483	0.493	5.805%	0.000 - 185.185%	4.612%	0.000 - 200.000%
Cortical Nucleus	0.766	0.687	0.668	0.670	1.940%	0.000 - 66.667%	1.036%	0.000 - 63.830%
Accessory Basal Nucleus	0.954	0.957	0.926	0.926	0.268%	0.000 - 24.442%	0.591%	0.000 - 18.502%
Paralaminar Nucleus	0.666	0.613	0.586	0.588	0.576%	0.000 - 60.606%	0.501%	0.000 - 103.704%
Corticoamygdaloid Transition	0.929	0.925	0.829	0.823	0.405%	0.000 - 20.930%	0.271%	0.000 - 18.568%
Anterior Amygdaloid Area	0.489	0.427	0.745	0.750	0.370%	0.000 - 189.744%	0.473%	0.000 - 190.909%

Additional file Table S2: Intraclass Correlation Coefficients (ICCs), Dice Coefficients, Bland–Altman bias as a portion of a volume's structure (Bias as POV), and Bland-Altman bias ranges for Amygdala Subnuclei for left and right hemisphere regions (e.g., ICC LH = Intraclass Correlation Coefficients for left hemisphere; Dice RH = Dice Coefficient for right hemisphere). Color coding is in accordance with excellent [green], good [yellow], poor [red] scores for ICCs and Dice Coefficients (ICC: 0.90-1.00 [excellent], 0.75-0.89 [good], 0.00-0.74 [poor]; Dice Coefficients: 0.70-1.00 [excellent], 0.50-.69 [good], 0-0.49 [poor]). We have also highlighted regions with >1% bias as a portion of a volume's structure in yellow. Values were derived using Freesurfer 7 longitudinal processing stream.

Spatial Reliability Differences in Relation to Person-level and MR-acquisition Factors Using Longitudinal Processing Stream

We next examined associations between spatial reliability (derived via longitudinal processing) and subject-level variables. Correlations between the Hippocampal-subfield Dice coefficients and subject-level variables are shown in Table S3. Differences in image quality were most significantly and negatively related to volumes in a majority of the hippocampal subfields at the $p < 0.05$ level (shown in Table S3). Sex, and to an even lesser extent, age, were significantly correlated with a few hippocampal subfield volumes.

Table S3.

Region	MRIQ r Dice		Sex r Dice		Age r Dice	
	LH	RH	LH	RH	LH	RH
Parasubiculum	-0.14	-0.08	-0.02	-0.01	0.04	0.04
Presubiculum Head	-0.21	-0.22	-0.05	-0.06	0.05	0.01
Presubiculum Body	-0.24	-0.25	-0.05	-0.03	0.001	0.06
Subiculum Head	-0.25	-0.06	-0.05	-0.03	-0.03	0.001
Subiculum Body	-0.20	-0.16	0.003	-0.01	0.00	0.06
CA1 Head	-0.27	-0.25	0.002	-0.04	-0.01	-0.01
CA1 Body	-0.08	-0.14	-0.05	0.002	0.06	-0.01
CA3 Head	-0.18	-0.15	-0.02	-0.12	0.06	0.04
CA3 Body	-0.13	-0.23	-0.01	-0.03	0.08	0.04
CA4 Head	-0.22	-0.22	-0.01	-0.03	0.02	0.01
CA4 Body	-0.12	-0.21	-0.02	-0.02	0.05	0.02
GC ML DG Head	-0.23	-0.18	0.00	-0.05	0.002	0.01
GC ML DG Body	-0.17	-0.22	-0.02	-0.07	0.05	0.03
Molecular Layer HP Head	-0.26	-0.21	0.01	-0.08	0.02	0.01
Molecular Layer HP Body	-0.17	-0.24	0.02	-0.04	0.03	0.03
Fimbria	-0.20	-0.24	0.08	0.02	0.08	-0.02
Hippocampal Fissure	-0.23	-0.20	-0.01	-0.11	0.02	0.03
Hippocampal Tail	-0.22	-0.24	-0.12	-0.00	0.03	-0.02

HATA	-0.18	-0.12	-0.03	-0.12	-0.03	0.04

Additional file Table S3: Correlation coefficient for bivariate correlations between Hippocampal Subfield Dice Coefficients and subject-level covariates: MRI Quality (Difference Score; MRIQ), Sex, and Age. These were completed for left hemisphere (LH) and right hemisphere (RH). Correlations with $p<0.05$ are highlighted in yellow. Subfield Abbreviations include: Cornu Ammonis (CA), Granule Cell and Molecular Layer of Dentate Gyrus (GC-ML-DG); Hippocampus-Amygdala-Transition-Area (HATA); Hippocampal Parcellation (HP). Values were derived using Freesurfer 7 longitudinal processing stream.

Correlations between spatial reliability and subject-level variables for the amygdala nuclei are reported in Table S4. Image quality was significantly and negatively related to several amygdala nuclei bilaterally including the lateral nucleus, the basal nucleus, the accessory basal nucleus, the corticoamygdaloid transition, and the anterior amygdaloid area (at $p<0.05$, see Table S4). These correlations indicate that image quality is more related to spatial reliability in amygdala regions when the longitudinal processing pipeline is used, compared to the cross-sectional pipeline. The spatial reliability of a minority of regions was also significantly and associated with sex; we observed no significant associations between age and image quality in longitudinally-derived metrics of reliability.

Table S4.

Region	MRIQ r Dice		Sex r Dice		Age r Dice	
	LH	RH	LH	RH	LH	RH
Lateral Nucleus	-0.20	0.22	-0.09	-0.05	0.04	0.02
Basal Nucleus	-0.18	-0.14	-0.02	-0.05	0.00	0.05
Central Nucleus	-0.06	-0.09	0.05	0.01	-0.00	0.03
Medial Nucleus	-0.05	-0.05	-0.02	-0.13	0.00	0.02
Cortical Nucleus	-0.04	-0.01	0.00	0.00	0.01	0.04
Accessory Basal Nucleus	-0.10	-0.13	-0.00	0.03	0.01	0.04
Paralaminar Nucleus	-0.19	-0.06	0.02	0.02	0.029	0.04
Corticoamygdaloid Transition	-0.19	-0.17	-0.04	-0.08	0.06	0.05
Anterior Amygdaloid Area	-0.12	-0.15	0.00	-0.05	0.06	0.04

Additional file Table S4: Correlation coefficient for bivariate correlations between Hippocampal Subfield Dice Coefficients and subject-level covariates: MRI Quality (Difference Score; MRIQ), Sex, and Age. These were completed for left hemisphere (LH) and right hemisphere (RH). Correlations with $p<0.05$ are highlighted in yellow. Values were derived using Freesurfer 7 longitudinal processing stream.

Volumes of Hippocampal Subfields and Amygdala Subnuclei

Supplemental Tables 5 and 6 display the average volumes per each subregion for hippocampal subfields (Table S5) and amygdala subnuclei (Table S6) in millimeters cubed (mm3).

Table S5.

Region	Mean LH (mm3)	SD LH (mm3)	Mean LH (mm3)	SD RH (mm3)
Parasubiculum	58.177	11.861	56.730	11.647
Presubiculum Head	145.911	21.557	139.207	17.880
Presubiculum Body	195.045	31.730	174.494	30.068
Subiculum Head	175.739	25.808	168.611	21.784
Subiculum Body	220.748	32.603	204.475	26.823
CA1 Head	610.509	82.839	645.382	84.665
CA1 Body	112.928	24.457	124.537	24.619
CA3 Head	119.278	22.007	132.774	23.703
CA3 Body	89.443	22.104	106.334	25.281
CA4 Head	133.301	18.223	142.190	19.196
CA4 Body	125.232	15.256	129.543	16.879
GC ML DG Head	153.987	21.788	165.679	23.135
GC ML DG Body	129.832	15.375	133.440	15.210
Molecular Layer HP Head	317.304	38.619	326.809	37.008
Molecular Layer HP Body	211.265	25.768	221.750	28.094
Fimbria	75.177	19.106	69.673	17.413
Hippocampal Fissure	125.652	32.431	147.475	33.396
Hippocampal Tail	602.868	82.474	628.700	81.715
HATA	67.314	11.869	72.912	12.926

Additional file Table S5: Mean and standard deviation (SD) volume estimates across three scans for each Hippocampal Subfield (LH = Left Hemisphere, RH = Right Hemisphere) in millimeters cubed (mm3). Subfield abbreviations include: Cornu Ammonis (CA), Granule Cell and Molecular Layer of Dentate Gyrus (GC-ML-DG); Hippocampus-Amygdala-Transition-Area (HATA); Hippocampal Parcellation (HP). Volume estimates derived using the FreeSurfer 7 longitudinal processing pipeline.

Table S6.

Region	Mean LH (mm3)	SD LH (mm3)	Mean LH (mm3)	SD RH (mm3)
Lateral Nucleus	703.861	85.709	726.398	84.255
Basal Nucleus	381.172	44.789	406.082	45.613
Central Nucleus	45.166	9.002	49.432	9.010
Medial Nucleus	8.149	4.465	8.972	4.636
Cortical Nucleus	22.509	5.364	24.912	4.733
Accessory Basal Nucleus	293.489	40.942	322.167	42.724
Paralaminar Nucleus	35.430	5.513	35.700	4.984
Corticoamygdaloid Transition	173.944	23.821	185.766	24.465
Anterior Amygdaloid Area	28.963	6.459	31.781	6.273

Additional file Table S6: Mean and standard deviation (SD) volume estimates across three scans for Amygdala Subnuclei (LH = Left Hemisphere, RH = Right Hemisphere) in millimeters cubed (mm3). Volume estimates derived using the FreeSurfer 7 longitudinal processing pipeline.
